# Prognostic Impact of Tumor-Associated Macrophages on Long-Term Oncologic Outcomes in Colorectal Cancer

**DOI:** 10.3390/life11111240

**Published:** 2021-11-16

**Authors:** Hyeong Chan Shin, Incheol Seo, Hasong Jeong, Sang Jun Byun, Shin Kim, Sung Uk Bae, Sun Young Kwon, Hye Won Lee

**Affiliations:** 1 Department of Pathology, Keimyung University School of Medicine, Dongsan Hospital, Daegu 42601, Korea; chan@dsmc.or.kr (H.C.S.); 4929292jhs@gmail.com (H.J.); pathol72@gmail.com (S.Y.K.); 2 Department of Microbiology, Dongguk University College of Medicine, Gyeongju 38067, Korea; htr@daum.net; 3 Department of Radiation Oncology, Keimyung University School of Medicine, Dongsan Hospital, Daegu 42601, Korea; kryph@dsmc.or.kr; 4 Department of Immunology, Keimyung University School of Medicine, Daegu 42601, Korea; god98005@dsmc.or.kr; 5 Department of Surgery, Keimyung University School of Medicine, Dongsan Hospital, Daegu 42601, Korea; sabiston0000@hanmail.net

**Keywords:** tumor-associated macrophages, colorectal cancer, tumor microenvironment, prognosis

## Abstract

This study evaluated the correlation between tumor-associated macrophages (TAMs) and long-term oncologic outcomes in colorectal cancer (CRC). We evaluated TAMs based on the expression of CD68, CD11c, and CD163 as optimal markers via immunohistochemistry in 148 patients with CRC who underwent surgical resection between September 1999 and August 2004. A high proportion of CD68-positive macrophages were associated with the occurrence of distant metastasis. A low proportion of CD11c-positive macrophages were associated with unfavorable overall survival (OS) and disease-free survival. CD11c-positive macrophages were found to act as independent prognostic factors for OS. An analysis of our long-term data indicated that TAMs are significantly associated with OS and prognosis in CRC.

## 1. Introduction

Colorectal cancer (CRC) is a common and malignant epithelial neoplasm prevalent in Korea and other advanced countries [1,2]. The identification of the predictors of disease recurrence and poor prognosis is critical for the successful treatment of patients with CRC and for discovering new therapeutic strategies. Recent studies on the tumor microenvironment have attracted attention for advancing the understanding of cancer biology and the identification of significant therapeutic targets. Macrophages, a major component of the tumor microenvironment, play an important role in the initiation and progression of various solid tumors [3,4,5]. CRC carcinogenesis is mediated by epigenetic and genetic changes in tumor cells and is also affected by tumor–host interactions. CRC tumors are associated with a dynamic immune response. Therefore, studies on tumor-associated macrophages (TAMs), that are known to be associated with prognosis and tumor growth, may provide predictive markers and identify indicators of tumor aggressiveness [6].

TAMs are abundantly clustered around tumor nests. The functional states of macrophages are classified as M1 and M2 phenotypes [7]. M1 macrophages are activated by helper T cell-related cytokines and bacterial products; they display pro-inflammatory activity and are tumoricidal. M2 macrophages release anti-inflammatory cytokines that contribute to the establishment of a tumor inflammatory microenvironment [8].

Several specific expression markers have been identified for the evaluation of TAMs. CD68 is a pan-macrophage marker that is commonly used in clinical practice. CD11c is considered a marker of M1 macrophages. CD163 is an optimal marker for M2 macrophages [9]. Recent studies have shown that TAMs play a crucial role in the tumorigenesis and progression of CRC and that they can be used as a potential target for cancer treatment [10,11,12]. However, the prognostic significance of TAMs in CRC is poorly understood. This study investigated the distribution patterns of macrophages in CRC using specific markers CD68, CD11c, and CD163 and possible associations with long-term oncologic outcomes.

## 2. Materials and Methods

### 2.1. Patients and Tissue Samples

Formalin-fixed paraffin-embedded (FFPE) block specimens were obtained using surgically resected primary tumors from 148 patients with CRC who underwent curative surgery and adjuvant chemotherapy at Keimyung University Dongsan Medical Center (Daegu, Korea) between September 1999 and August 2004. The exclusion criteria were cancer-related to inherited syndromes, synchronous malignancies, locally advanced rectal cancer that underwent neoadjuvant chemoradiation and patients who were lost before follow-up.

The baseline clinicopathological characteristics and clinical outcome data were collected retrospectively from the Colorectal Cancer Database of the Department of Colorectal Cancer Surgery, and the Pathological Diagnosis Database of the Department of Pathology. The researchers reviewed all the available medical records related to CRC and extracted clinical information including the American Joint Committee on Cancer (AJCC) primary tumor, lymph node, distant metastasis (TNM) classification, the numbers of positive and negative lymph nodes harvested, and tumor location, and determined the cause of death in deceased individuals. This prospective observational study was approved by the Institutional Review Board of Keimyung University and Dongsan Medical Center (IRB No. 2016-08-020).

### 2.2. Evaluation Parameters

The eighth edition of the AJCC classification system was used to determine the pathological primary tumor depth (pT), the number of metastatic regional lymph nodes (pN), and the cancer stage [13]. A postoperative clinical examination, measurement of serum carcinoembryonic antigen (CEA) levels, chest radiography every three months, and computed tomography of the chest and abdomen every six months were performed at each follow-up examination over three years. After three years, the follow-up interval for measuring serum CEA levels and performing chest radiography was changed to six months. Recurrence was defined as the presence of histologically proven or radiologically confirmed tumors, and the location of recurrence was defined as the site of first recurrence after complete resection. Local recurrence was defined as any tumor recurrence in the surgical field, and synchronous systemic recurrence with local recurrence included systemic recurrence. The overall survival (OS) time was defined as the time from the date of surgery to the date of the most recent follow-up visit or death from any cause, and the disease-free survival (DFS) time was defined as the time from surgery to the time of development of any type of recurrence. 

### 2.3. Tissue Microarray Construction

All of the human primary CRC tissue samples were prepared using FFPE blocks. The paraffin blocks, including representative tumor lesions, were selected after reviewing the commensurate hematoxylin and eosin-stained slides. Representative tumor lesions (1 to 3) from each case were marked on the source blocks and manually cored using a 3.0 mm diameter cylindrical device. Each selected core was then re-embedded into the recipient blocks.

### 2.4. Immunohistochemistry (IHC)

The proportion of infiltrating macrophages was determined based on the immunopositivity (CD68, CD11c, and CD163). Sections (4 μm thick slices) from the tissue microarrays were cut in a 10% formalin buffer, embedded in paraffin, mounted onto Superfrost Plus glass slides (VWR Scientific, West Chester, PA, USA), and incubated at 60 °C for 15 min. The slides were deparaffinized in xylene, rehydrated in graded alcohol solutions, and washed with tap water. Endogenous peroxidase activity was blocked by the addition of 3% H_2_O_2_. The slides were placed in a steam cooker filled with 10 mM sodium citrate buffer (pH 6.0) for antigen retrieval after treatment with a blocking agent (DAKO, Carpinteria, CA, USA) for 10 min to block nonspecific protein binding. Immunohistochemical staining for specific macrophage markers was performed using a BenchMark ULTRA automatic stainer (Ventana Medical Systems, Inc., Tucson, AZ, USA) according to the manufacturer’s instructions. The following primary antibodies were used in this study: CD68 (1:400; Santa Cruz Biotechnology, Dallas, TX, USA), CD11c (1:100; Abcam, Cambridge, UK), and CD163 (1:200; Thermo Fisher Scientific, Waltham, MA, USA). The bound antibodies were visualized using an ultraView Universal DAB Detection Kit (Roche, Basel, Switzerland). The slides were counterstained using Mayer’s hematoxylin. Slides that were incubated without primary antibodies were used as negative controls. The positive and negative controls were stained appropriately.

### 2.5. Assessment of IHC Staining for TAMs

Three fields containing the highest infiltration of IHC-stained cells were selected at 400× magnification and observed using an Aperio ImageScope (Leica Biosystems Imaging, Inc., Nussloch, Germany). The number of IHC-stained cells showing only monocytoid or macrophage-like morphology was counted, and the average of the three fields was calculated. For the statistical evaluation, the average numbers of positive cells were assigned to high and low groups based on the cut-off point.

### 2.6. Statistical Analyses

Statistical analyses were performed using SPSS version 18.0 (IBM Corp., Armonk, NY, USA) and R 3.5.3 (R Development Core Team, https://www.R-project.org, accessed on 15 October 2021; Vienna, Austria) [14]. An independent *t*-test was performed to analyze the association between the number of macrophages (CD68-, CD11c-, and CD163-positive) and the clinicopathological parameters. The Maxstat method in R 3.5.3 was used to identify the cut-off points for the number of CD68-, CD11c-, and CD163-positive macrophages. The cut-off points for the number of CD68-, CD11c-, and CD163-positive macrophages in OS were 104.7, 60.0, and 64.5, respectively. The cut-off points for the number of CD68-, CD11c-, and CD163-positive macrophages in DFS were 29.3, 101.0, and 21.0. Both a Kaplan-Meier analysis and log-rank tests were performed to demonstrate the difference between OS and DFS based on CD68, CD11c, and CD163 expression. Cox proportional hazard models were used to determine the hazard ratios (HRs) for death from CRC or other causes based on the CD68, CD11c, and CD163 expressions in both univariate and multivariate analyses. A *p*-value of <0.05 was considered significant.

### 2.7. Survival Analysis Using Public CRC Datasets

To evaluate the prognostic value of TAMs for predicting the survival in patients with CRC, we analyzed six public datasets from the Gene Expression Omnibus and The Cancer Genome Atlas (TCGA) databases, along with the datasets GSE17536, GSE17537, GSE33113, GSE39582, GSE41258, and COAD. A total of 1,530 patients with primary CRC for whom survival data were available were selected for analysis. CIBERSORT was used to deconvolute the abundance of TAMs, and M1 and M2 macrophages from the mRNA expression datasets [15]. A source code for the CIBERSORT software was provided by the developer and was executed in R using the immunedeconv package [16]. Probe identifiers of microarray data were converted to Hugo Gene Nomenclature Committee gene symbols. In order to convert multiple probe identifiers to one gene symbol, only the ID with the greatest expression of FPKM and TCGA expression data was converted to TPM. CIBERSORT was executed in its “absolute mode” with disabled quantile normalization options and without permutation. A leukocyte gene signature matrix (LM22) was used as a reference to distinguish between infiltrating macrophages in general and the M1 and M2 subtypes. A Kaplan-Meier survival analysis was performed based on the data of TAMs, M1, and M2 obtained using CIBERSORT. The cases were divided into two groups according to the median abundance of TAMs, M1, or M2.

## 3. Results

### 3.1. Characteristics of the Patients

Among the 148 patients, 92 (62.2%) were males and 56 (37.8%) were females. According to the eighth edition of AJCC TNM staging, 1.4% (2/148) of cases were classified as pT1, 17.6% (26/148) of cases were pT2, 73.6% (109/148) of cases were pT3, and 7.4% (11/148) cases were pT4. Among the patients, 70 (47.3%) were classified as N0, 41 (27.7%) as N1, and 37 (25.0%) as N2. Fifteen patients (10.1%) were classified as M1. According to differentiation, 3.4% (5/148) of the cases were well differentiated, 89.9% (133/148) were moderately differentiated, 4.1% (6/148) were poorly differentiated, and 2.7% (4/148) showed mucinous differentiation. The age of the patients ranged from 32–92 years (mean, 67.2 years).

### 3.2. Clinical Significance of Macrophage Infiltration

**Figure 1:**
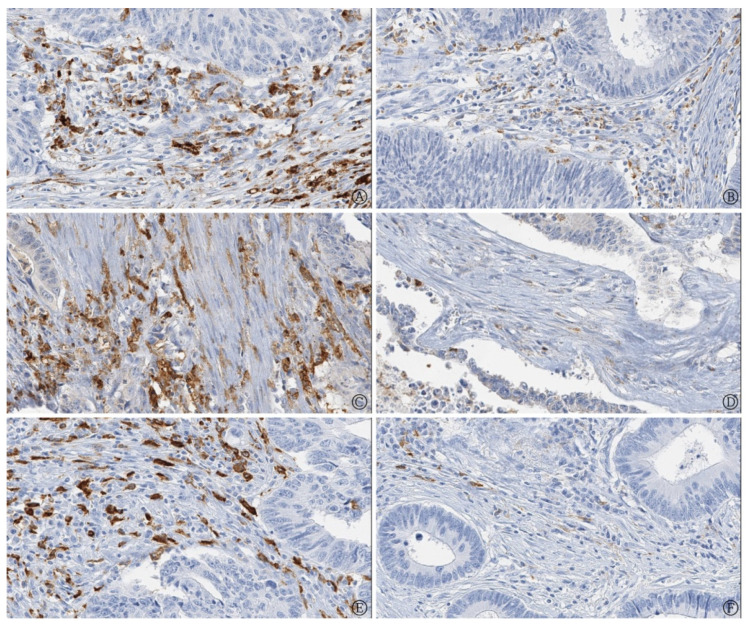
Immunohistochemistry for tumor-associated macrophages in colorectal cancer (**A**) Higher number of CD68 immunoreactive macrophages (**B**) Lower number of CD68 immunoreactive macrophages (**C**) Higher number of CD11c immunoreactive macrophages (**D**) Lower number of CD11c immunoreactive macrophages (**E**) Higher number of CD163 immunoreactive macrophages (**F**) Lower number of CD163 immunoreactive macrophages (400× magnification).

The CD68, CD11c, and CD163 expressions were evaluated for all of the 148 samples (Figure 1). The relationship between the number of macrophages (CD68-, CD11c-, and CD163-positive macrophages) and clinicopathological parameters of the patients is shown in Table 1. A high number of CD68-positive macrophages was associated with the presence of distant metastasis. There were no significant differences in other parameters.

### 3.3. Prognostic Significance of CD68-, CD11c-, and CD163-Positive Macrophages

**Figure 2:**
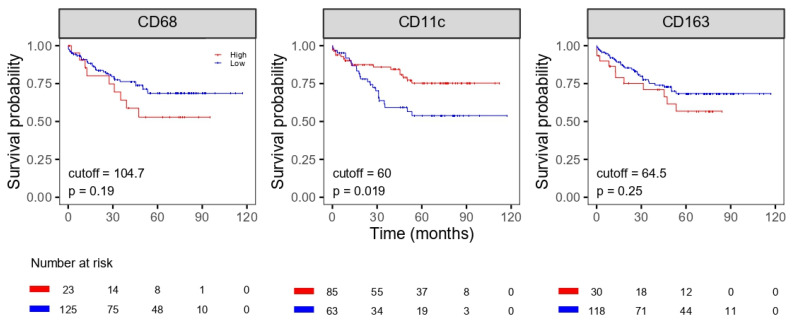
Overall survival of patients with colorectal cancer according to densities of macrophages.

**Figure 3:**
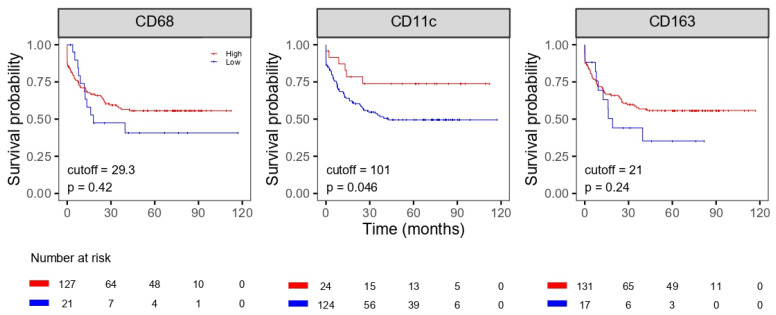
Disease-free survival of patients with colorectal cancer according to densities of macrophages.

We found that infiltration with a low number of CD11c-positive macrophages correlated with an unfavorable OS and DFS (*p* = 0.019 for OS and *p* = 0.046 for DFS); however, the number of CD68- and CD163-positive macrophages did not correlate with OS and DFS (Figure 2 and Figure 3).

In the univariate Cox regression analysis, distant metastasis and the number of CD11c-positive macrophages were the independent prognostic factors for OS. Lymph node metastasis was an independent prognostic factor for DFS (Table 2).

In the multivariate analysis, lymph node metastasis, distant metastasis, and the number of CD11c-positive macrophages were the independent prognostic factors for OS (Table 3).

### 3.4. Public Dataset Analysis

**Figure 4:**
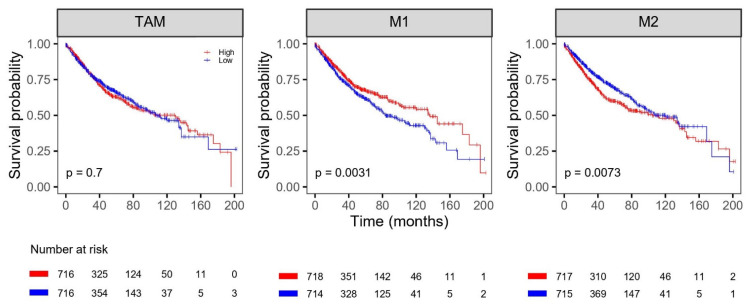
Kaplan-Meier analysis of overall survival with proportion of TAM, M1 and M2 in the public database cohort. TAM = tumor-associated macrophage.

**Figure 5:**
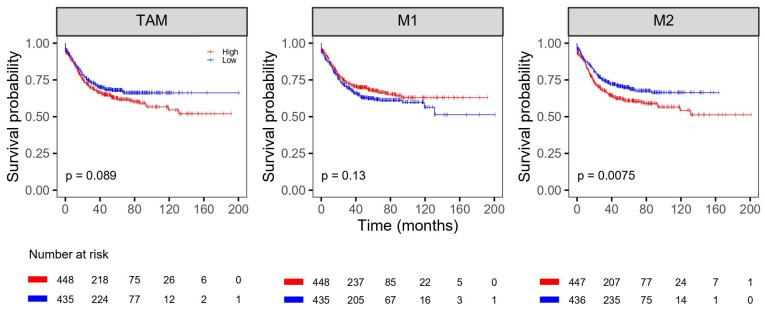
Kaplan-Meier analysis of disease-free survival with proportion of TAM, M1 and M2 in the public database cohort. TAM = tumor-associated macrophage.

The univariate survival analysis results for the six public CRC datasets are shown in Figure 4 and Figure 5 (Supplementary Figures S1 and S2). The OS and DFS were analyzed in 1432 subjects and 883 subjects for a total of 1530 subjects, respectively. High levels of M1 macrophages were associated with better OS (*p* = 0.0031). Low levels of M2 macrophages were associated with better OS and DFS (*p* = 0.0073 and 0.0075, respectively). A significant association was observed for TAM abundance. Also, the characteristics of the public dataset using in this analysis is shown in Supplementary Table S1.

## 4. Discussion

TAMs are leukocyte member present in the tumor microenvironment. Recently, it was found that these macrophages can be polarized into two macrophage types (M1 and M2). Macrophages found in the tumor microenvironment are often referred to as TAMs, which mostly exhibit the M2 phenotype [17]. TAMs usually interfere with tissue homeostasis and promote tumor growth, progression, invasion, and metastasis. Therefore, TAMs are primarily associated with a poor prognosis of tumors; however, a few studies have reported conflicting results [18,19]. 

We evaluated TAM, M1, and M2 proportions in CRC using the following three markers: CD68, CD11c, and CD163, respectively. Various types of macrophages are distributed in the surrounding matrix, adjacent to malignant tumors. The proportion of TAM types varied according to each case. Our data revealed that patients with a high level of CD11c-positive macrophage infiltration had favorable survival, and CD11c-positive macrophages were an independent prognostic factor for OS. 

Similarly, the multivariate analysis suggested that this factor represents a prognostic factor in patients with CRC. The infiltration of CD68-and CD163-positive macrophages was correlated with poor OS, whereas high proportions of CD68- and CD163-positive macrophages were associated with favorable DFS. These results are significant. We validated the results using public data sets. Since the data sets were obtained from bulk tissues, each macrophage subtype was analyzed via a deconvolution analysis instead of using a single marker. Similar results were obtained by using these publicly available CRC cohort datasets.

Previous studies have evaluated TAMs in various human malignancies, including breast cancer, CRC, pancreatic cancer, and non-small cell lung cancer [6,19,20,21]. The clinical outcome may vary depending on the proportion of TAMs that exhibit conflicting functions. Therefore, it is important to study the characteristics of TAMs as a treatment target. 

Unlike previous findings, the density of a CD68-positive TAM was not related to survival in CRC patients in our study. Additionally, Nitric oxide synthase (iNOS), another M1 subset marker, TAM, was not associated with survival, but our results showed that CD11c-positive TAM was significantly associated with OS and DFS [22,23]. To our knowledge, CD11c is yet to be evaluated as a TAM marker in CRC. CD11c is a marker that has been traditionally associated with dendritic cells, but a recent study found that it is expressed by some macrophages. TAMs share certain characteristics with dendritic cells, infiltrated sites and the secretion of similar pro-inflammatory cytokines [24]. Furthermore, Gulubova et al. revealed that transforming growth factor (TGF)-β1 and interleukin (IL)-10 are associated with the tumorigenesis and prognosis of CRCs [25]. The prognostic value of CD11c-positive M1 macrophage has been elucidated in hepatocellular carcinoma [26] and breast cancer [27].

TAMs have been shown to express immune checkpoint markers [28]. Furthermore, the results of this study can be applied to the analysis of programmed death-ligand 1 (PD-L1) markers. Immune checkpoints represent the major defense system of tumors against the antitumor immunity of the host and play an important role in suppressing T cell-mediated immune responses in the tumor microenvironment. Currently, PD-L1 immunohistochemical staining markers are analyzed without distinguishing between immune cells that are composed of lymphocytes, dendritic cells, and macrophages. Considering the function and crucial role of TAMs as well as the PD-1/PD-L1 axis in tumor progression, PD-L1 expression should be evaluated via the classification of the types of immune cells and macrophages using dual staining.

Our study has several limitations. Since limited tissue microarrays were used in the analyses, it was difficult to distinguish between the intratumoral and invasive front regions. Additional analyses are required to evaluate the microsatellite status. The present study may help to clarify the relationship between tumor aggressiveness and the detailed tumor microenvironment. Based on our results, TAMs may represent a novel therapeutic target and predictive factor for CRC. 

## 5. Conclusions

In conclusion, our study showed that TAMs in the tumor microenvironment serve as a prognostic factor for CRC. Given that the focus on immunotherapy and immunologic reactions has recently increased, our study on TMAs provides a relevant contribution to the literature.

## Figures and Tables

**Table 1:** Clinicopathologic parameters of patients and results of mean values of macrophages (CD68, CD11c and CD163).

Clinicopathologic Features	n = 148	CD68 Positive		CD11c Positive		CD163 Positive	
		Mean ± SD	*p*-Value	Mean ± SD	*p*-Value	Mean ± SD	*p*-Value
Age (mean)	32–92 (67.2)						
Gender							
Male	92	63.36 ± 31.83	0.31	66.32 ± 30.60	0.87	42.6 ± 23.07	0.33
Female	56	76.07 ± 38.27		75.34 ± 31.27		51.07 ± 25.77	
T Stage							
1, 2	28	67.40 ± 36.36	0.9	72.94 ± 35.91	0.55	43.40 ± 21.22	0.6
3, 4	120	68.34 ± 34.62		68.98 ± 29.93		46.10 ± 25.16	
LN metastasis							
Absent	70	68.00 + 36.81	0.96	70.15 ± 32.22	0.88	44.13 ± 20.92	0.49
Present	78	68.32 ± 33.20		69.35 ± 30.19		46.90 ± 27.25	
Distant metastasis							
Absent	133	65.81 ± 33.78	0.01	69.40 ± 32.07	0.7	45.07 ± 23.80	0.44
Present	15	89.02 ± 38.26		72.69 ± 20.62		50.23 ± 29.90	
Differentiation							
Well and moderate	138	68.02 ± 33.75	0.85	70.09 ± 31.62	0.61	45.48 ± 24.41	0.84
Poorly and mucinous	10	70.23 ± 49.75		64.80 ± 22.64		47.1 ± 25.89	

SD = Standard deviation; LN = lymph node.

**Table 2:** Univariate Cox regression analysis for OS and DFS.

Variables	OS			DFS		
	HR	95% CI	*p*-Value	HR	95% CI	*p*-Value
Age	1.018	0.988–1.049	0.247	1.003	0.980–1.027	0.795
Gender (Male vs. Female)	0.815	0.410–1.617	0.558	0.772	0.441–1.351	0.364
T stage (stage 1, 2 vs. stage 3, 4)	2.231	0.653–7615	0.200	2.136	0.827–5.519	0.117
Lymph node metastasis (+ vs. −)	2.144	0.977–4.707	0.057	3.534	1.849–6.751	0.000
Distant metastasis (+ vs. −)	4.847	1.959–11.990	0.001	5.97 × 10^5^	0.000–3.00 × 10^73^	0.867
Differentiation (Well and moderate vs. poorly and mucinous)	2.089	0.599–7.285	0.248	1.884	0.767–4.630	0.167
CD68	1.005	0.994–1.017	0.362	1.003	0.993–1.013	0.550
CD11c	0.985	0.972–0.999	0.032	0.990	0.980–1.001	0.063
CD163	1.002	0.990–1.014	0.747	0.999	0.988–1.010	0.848

OS = Overall survival; DFS = Disease free survival; HR = Hazard ratio; CI = Confidence interval.

**Table 3:** Multivariate Cox regression analysis for OS.

Variables	OS		
	HR	95% CI	*p*-Value
Lymph node metastasis (+ vs. −)	2.425	1.139–5.167	0.022
Distant metastasis (+ vs. −)	4.464	2.087–9.548	0.000
CD11c	0.988	0.977–0.999	0.040

OS = Overall survival; HR = Hazard ratio; CI = Confidence interval.

## Supplementary Materials

The following are available online at https://www.mdpi.com/article/10.3390/life11111240/s1, Figure S1: Overall survival probability, Figure S2: Disease-free survival probability, Table S1: Characteristics of public dataset.
