# Supplementary material for: Prognostic Impact of Tumor-Associated Macrophages on Long-Term Oncologic Outcomes in Colorectal Cancer
Source: Life (Basel). 2021 Nov 16;11(11):1240. doi: 10.3390/life11111240 (PMC8618174; doi:10.3390/life11111240)
Supplement: Supplementary file 1 [file life-11-01240-s001.zip › life-1423301-supplementary.pdf]

Supplementary material

Table S1. Characteristics of public dataset.

	**GSE17536**	**GSE17537**	**GSE33113**	**GSE39582**	**GSE41258**	**TCGA-COAD**
Total N	177	55	96	585	390	456
Total N (primary CRC with clinical information)	177	55	90	566	186	456
N (OS)	177	55	0	562	185	453
N (DFS)	145	55	90	557	36	0
Age	65.48023	62.30909	70.39225	66.90602	63.45405	66.91939
Age SD	13.08195	14.35125	12.95312	13.26697	13.92407	13.07954
Gender						
F	81	29	48	256	87	216
M	96	26	42	310	98	243
Stage						
O	0	0	0	4	0	0
I	24	4	0	33	28	76
II	57	15	90	264	50	178
III	57	19	0	205	49	129
IV	39	17	0	60	58	65

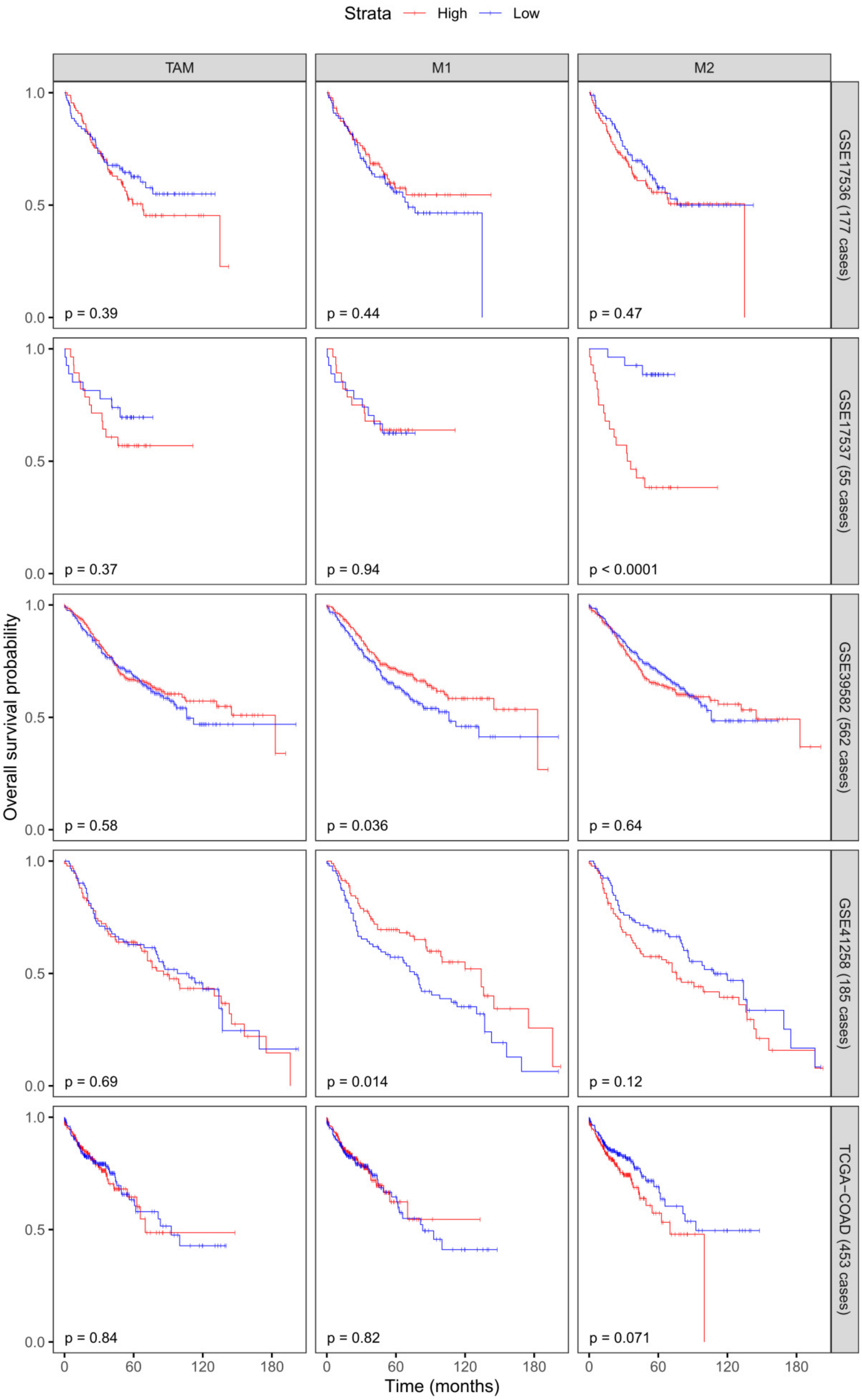

Figure S1. Overall survival probability.

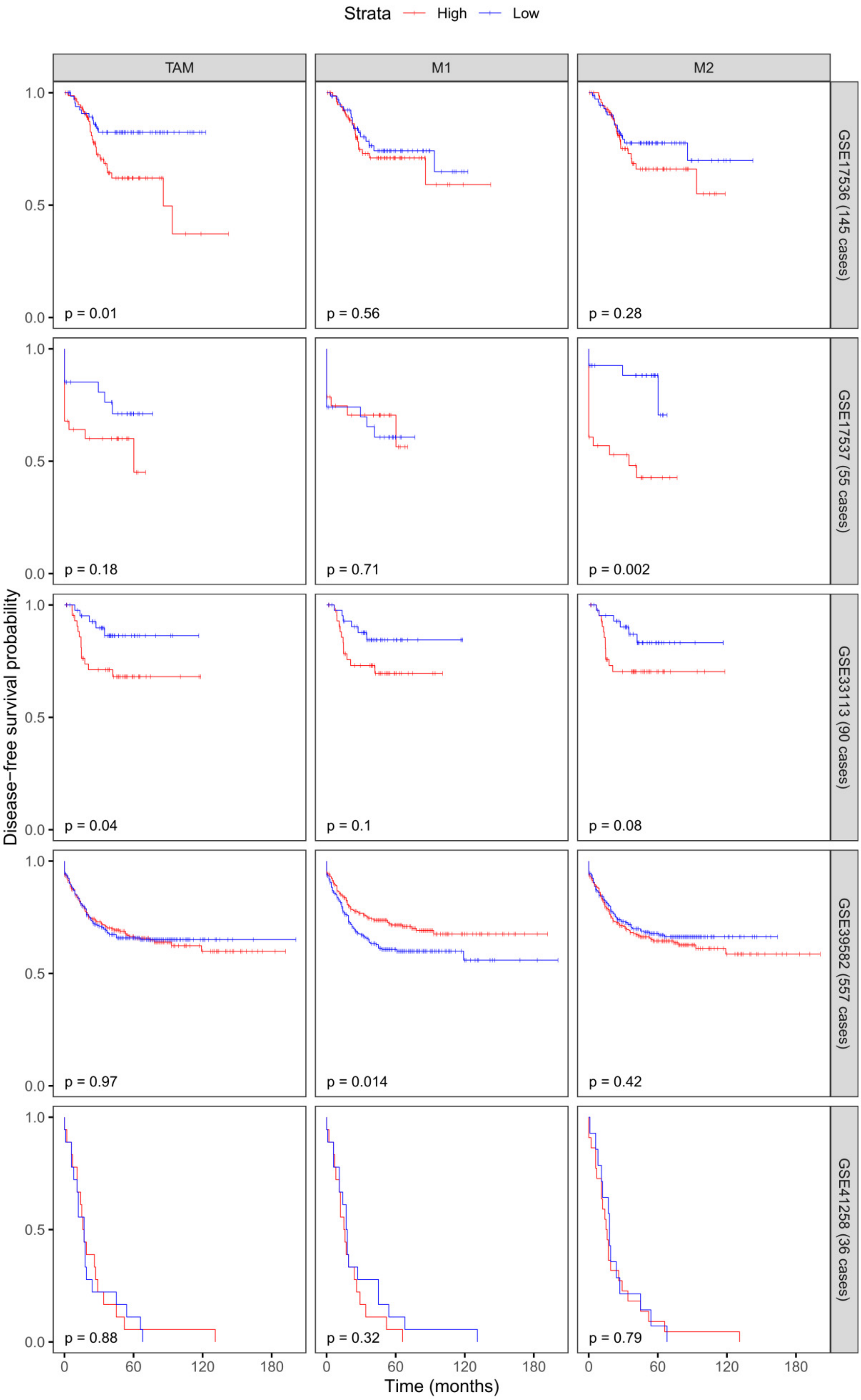

Figure S2. Disease-free survival probability.
